# The Antifungal Effects of Equol Against *Candida albicans* Involve Mitochondrial Dysfunction

**DOI:** 10.3390/jof11050339

**Published:** 2025-04-27

**Authors:** Anni Ge, Hao Zhou, Xi Yang, Chunling Zhao, Caiyan Xin, Zhangyong Song

**Affiliations:** 1 School of Basic Medical Sciences, Southwest Medical University, Luzhou 646000, China; 17852273573@163.com (A.G.); zhjdkl@126.com (H.Z.); yx18283865097@163.com (X.Y.); chunlingzhao7005@swmu.edu.cn (C.Z.); 2 The Public Platform of Molecular Biotechnology, Public Center of Experimental Technology, Southwest Medical University, Luzhou 646000, China; 3 Hemodynamics and Medical Engineering Combination Key Laboratory of Luzhou, Southwest Medical University, Luzhou 646000, China

**Keywords:** *Candida albicans*, Equol, antifungal agent, cell wall, cell membrane, mitochondrial dysfunction

## Abstract

Novel antifungal agents are urgently needed because of the increasing number of drug-resistant *Candida* strains encountered in clinical practice and the limited variety of available antifungal drugs. Equol, a metabolite of soy isoflavone glycosides, exhibits antifungal activities. In this study, Equol had good inhibitory activity against *Candida* species. The lowest inhibitory concentration of 125–500 μg/mL was confirmed by the gradient dilution method. In addition, transmission electron microscopy and the relative content assay showed that Equol altered the cell wall and membrane of *Candida albicans*. Further studies found that Equol treatment increased the intracellular levels of reactive oxygen species and Ca^2+^. Subsequent experiments suggested that Equol treatment depolarized the membrane potential of *C. albicans* and up-regulated the expression of the apoptosis-inducing factor gene. These results confirmed that Equol damaged the cell wall and membrane, dysregulated the intracellular components, induced oxidative stress and Ca^2+^ accumulation, and ultimately resulted in mitochondrial dysfunction. Collectively, these findings demonstrated that Equol is a potential antifungal agent.

## 1. Introduction

In 2022, the World Health Organization included *C. albicans* and *Candidozyma auris* among the four key priority fungal pathogens because of the increasing incidence of drug resistance [1,2]. In 2023, the proposed guidelines for the Joint Plan of Action for Health stated that antimicrobial drug resistance is the leading cause of death due to microbial infections [3]. *C. albicans*, a component of the normal human microbiota, is a diploid polymorphic yeast that resides on the skin and mucosal surfaces. The pathogenic potential of *C*. *albicans* has been linked to a state of compromised immunity due to critical illnesses, implanted medical devices, or during long-term use of broad-spectrum antibiotics [4]. *C*. *albicans* is considered an opportunistic pathogen that causes a range of diseases, including mucocutaneous candidiasis and recurrent candidal vaginitis, which accounts for 103–172 million cases annually [5]. Moreover, the incidence of systemic candidiasis usually ranges from about 2 to 21 cases per 100,000 population, with about 1.6 million cases of systemic infection and 99,500 deaths per year [6]. Typically, *C. albicans* accounts for about 40–60% of *Candida* isolates from hospitalized patients [7,8].

Due to the lack of new antifungal treatment options and related biocompatibility issues, antifungal resistance has further increased, limiting medical applications [9]. Therefore, there is an urgent need to develop novel, highly effective antifungal agents with low toxicity for treatment of diseases associated with pathogenic *Candida*. In fungi, cell walls and membranes are the primary line of protection against environmental stressors and medication harm [10,11,12]. These structures may experience adaptive changes or even destruction when subjected to such loads, which could have several negative effects, such as intracellular oxidative stress and calcium overload [13,14]. The excessive production of reactive oxygen species (ROS) can damage nucleic acids, proteins, and lipids, and increase the permeability of the cell membrane, ultimately resulting in mitochondrial-dependent programmed cell death [15,16,17]. Besides ROS, the excessive accumulation of intracytoplasmic Ca^2+^ disrupts mitochondrial membrane potential (MMP), causing mitochondrial dysfunction and the release of pro-apoptotic factors, which initiate mitochondria-dependent programmed cell death [18]. Thus, damage to cell walls and membranes by pathogenic fungi, along with substances that induce excessive ROS production and intracytoplasmic Ca^2+^ overaccumulation, are potential therapeutic approaches against fungal infections.

Equol is a metabolite of soy isoflavones under the action of the intestinal microflora [19]; it is easily absorbed by the colon wall and cleared more slowly in the plasma [20], and is most commonly taken orally at a dose of 10 mg per day by adults according to a US-based online medical information platform (https://www.webmd.com, accessed on 13 February 2025). It has been shown to exhibit pharmacological activities against inflammation, tumor cell proliferation, and bacterial infection [21,22,23]. For instance, Equol was reported to inhibit biofilm formation and decrease the motility of *Yersinia enterocolitica* [24], inhibit the growth and sporulation of *Clostridioides difficile* in a concentration-dependent manner [23], and block the production of carbapenemases and virulence factors of carbapenem-resistant *Escherichia coli* when used in combination with meropenem [22]. Moreover, Equol can effectively inhibit mycelial growth, conidial production, and the germination of the plant pathogenic fungus *Magnaporthe oryzae* and reduce leaf virulence of rice and barley [25]. However, although Equol activity against *C. albicans* has been investigated [26,27], the specific antifungal mechanism remains unclear. In view of the wide range of biological effects mediated by Equol and previous work by our group to clarify the efficacy against *C. albicans* infection in vivo and ex vivo [27], the aim of the present in-depth investigation was to elucidate the antifungal mechanism of Equol against *C. albicans* as a novel antifungal agent.

## 2. Materials and Methods

### 2.1. Chemicals and Reagents

Equol and amphotericin B (AmB) were purchased from Shanghai Yuanye Bio-Technology Co., Ltd. (Shanghai, China). Roswell Park Memorial Institute (RPMI) 1640 medium was purchased from HyClone Laboratories, Inc. (South Logan, UT, USA). Calcofluor white (CFW) and Congo red dye were purchased from Sigma-Aldrich Corporation (St. Louis, MO, USA). Prior to each experiment, a stock solution of Equol dissolved in dimethyl sulfoxide at 50 mg/mL was prepared and diluted with YPD medium (1% yeast extract, 2% peptone, 2% glucose) to the target concentration.

### 2.2. Strains and Culture Conditions

Standard strains purchased from the American Type Culture Collection (ATCC; Manassas, VA, USA) included *Candida albicans* ATCC MYA-2876, *Candida parapsilosis* ATCC 22019, and *Cryptococcus neoformans* ATCC 208821. In addition, clinical isolate strains of *Candidozyma auris* (named swcau and swcau1) and *Candidozyma duobushaemulonii* (named swcd) were obtained by routine hospital microbiology isolation procedures for experimental studies. All strains were stored in 15% glycerol at −80 °C and incubated overnight in YPD medium at 37 °C and 200 rpm before use.

### 2.3. Determination of Minimal Inhibitory Concentration (MIC)

The MIC of Equol against the tested strains was determined in reference to the American Society for Clinical and Laboratory Standard M27-A4. Briefly, the fungi were activated and resuspended in RPMI-1640 medium to a final concentration of 0.25–5 × 10^3^ cells/mL. Equol at 1–1024 μg/mL was added to the drug-treated wells (CTWs), positive control wells (PCWs) with no drug, and sterile blank control wells (BCWs) of 96-well plates, with three replicates/group. The plates were incubated for 24 h at 35 °C, and *C. neoformans* was incubated for 72 h. Afterward, the optical density (OD) of each well was measured at 600 nm using a multifunctional microplate reader. The inhibition rate was calculated as 100 × [1 − (OD_CTW_ − OD_BCW_)/(OD_PCW_ − OD_BCW_)]. The MIC was defined as the concentration that resulted in 90% growth inhibition as compared to the control.

### 2.4. Growth Inhibition Kinetics

Growth curves were generated as described in a previous study [28]. Briefly, *C. albicans* ATCC MYA-2876 cells cultured overnight were diluted to 5 × 10^5^ cells/mL in YPD medium containing either 250 µg/mL Equol or 2 µg/mL AmB. Controls were free of Equol and AmB. Cells were incubated at 37 °C and 200 rpm. The OD at 600 nm was measured during co-cultivation using a multifunctional microplate reader. Growth kinetics were recorded for 48 h.

### 2.5. Effects of Equol on Proliferation of Ana-1 Macrophages Cell

The Ana-1 cell line was counted to 1 × 10^5^ cells/mL, and 100 μL of the cell suspension was added to each well of a 96-well cell culture plate. The plate was incubated overnight at 37 °C in a 5% CO_2_ incubator. After discarding the culture medium, 0–500 μg/mL of Equol was added. Following incubation, cells were washed twice with phosphate-buffered saline (PBS), and 100 μL of 10% Cell Counting Kit-8 reagent (APExBIO Technology, Houston, TX, USA) was added. The plate was incubated for 2 h, and the absorbance at 450 nm was measured using a microplate reader. The cell survival rate (%) was calculated as (OD administration − OD blank)/(OD control − OD blank) × 100%. The experiment was repeated three independent times.

### 2.6. Measurement of Cell Membrane Permeability

The membrane permeability of *C. albicans* ATCC MYA-2876 cells was quantified and visualized using the fluorescence intensity method and fluorescence microscopy, respectively. Membrane integrity was assessed by staining with propidium iodide (PI). Overnight cultured cells were diluted to 1 × 10^6^ cells/mL in YPD medium and incubated for 8 or 12 h with 250 µg/mL Equol at 37 °C with 200 rpm. Untreated cells were used as a negative control. After incubation, the fungal cells were adjusted to 1 × 10^6^ cells/mL with PBS, followed by the addition of 10 mM PI (Sigma-Aldrich Corporation) solution and incubation for 15 min at 37 °C. After washing with PBS, membrane permeability was evaluated by measuring the fluorescence intensity of PI at excitation and emission wavelengths of 525 and 590 nm, respectively, using a multimode microplate reader (Varioskan™ LUX; Thermo Fisher Scientific, Waltham, MA, USA) [29]. The samples were collected and observed under a fluorescence microscope at 40× magnification.

### 2.7. Cell Wall Staining and Microscopic Examination

The fluorescent brightener and aniline blue staining methods have been applied to assay the cell walls of *C. albicans* and *C. neoformans.* Based on these established methods [29,30], the effect of Equol on the cell wall of *C. albicans* ATCC MYA-2876 was determined. Briefly, fungal cells were incubated at 200 rpm at 37 °C for 8 or 12 h in the presence or absence of 250 μg/mL Equol, then washed with PBS, resuspended to 5 × 10^6^ cells/mL, fixed with 4% paraformaldehyde for 15 min at room temperature, washed again with PBS, and incubated with 5 μg/mL CFW at 37 °C in the dark for 10 min for the staining of chitin. Afterward, the cells were washed twice with PBS and the total chitin content was quantified by measuring fluorescence with a microplate reader at excitation and emission wavelengths of 365 and 435 nm, respectively. In addition, the cells were observed under a fluorescence microscope at 40× magnification (BX63; Olympus Corporation, Tokyo, Japan). For quantification of total β-1,3-glucan, the cells were incubated with 0.1% aniline blue (Wako Pure Chemical Industries, Ltd., Osaka, Japan) at 80 °C for 15 min. Subsequently, fluorescence was measured at excitation and emission wavelengths of 400 and 460 nm, respectively. Change in fluorescence (∆F) was calculated as [F(experimental group) − F(blank group)], where F(experimental group) is the fluorescence of the test samples, F(blank group) is the fluorescence of the test samples, and F(blank group) is the fluorescence of the test group without the dye solution.

### 2.8. Reverse Transcription Quantitative Polymerase Chain Reaction (RT-qPCR)

Overnight cultures of fungal cells (10^6^ cells/mL) were incubated with or without Equol at 37 °C and 200 rpm and collected by centrifugation at 12,000 rpm for 3 min at 4 °C. Total RNA was isolated using a Yeast Processing Reagent (TaKaRa, Dalian, China) and reverse-transcribed into complementary DNA using a PrimeScript™ RT kit with a gDNA Eraser (TaKaRa). RT-qPCR was performed using TB Green Premix Ex Taq™ II Master Mix (TaKaRa). qPCR data were first normalized with *β-actin* expression as an internal reference, and then relative expression levels were calculated using 2^−ΔΔCT^ at each time point with reference to the untreated group [27].

### 2.9. Measurement of ROS Concentrations

Dichlorodihydrofluorescein diacetate (DCFH-DA) was used to determine intracellular ROS levels. Overnight cultured fungal cells were diluted to 1 × 10^6^ cells/mL and cultured in 250 µg/mL of Equol in YPD medium for 8 or 12 h at 37 °C and 200 rpm. Untreated fungal cells were used as a negative control. After incubation, the test cells were resuspended in PBS to 1 × 10^6^ cells/mL, incubated with 40 μmol/L DCFH-DA for 30 min at 37 °C, and washed twice with PBS. Fluorescence intensity (FI) values were detected with an enzyme labeling instrument at excitation and emission wavelengths of 490 and 530 nm, respectively. The FI values, reflecting intracellular ROS levels, were calculated by subtracting the FI values of the stained cells from those of the unstained cells [29]. The cells were collected and observed under a fluorescence microscope at 40× magnification.

### 2.10. Measurement of Cytosolic Ca^2+^ Concentrations

The Fluo-3/4 AM probe method for measuring intracellular Ca^2+^ concentration has been widely used in *C. albicans* and *C. neoformans* [17,31]. Additionally, Fluo-4 AM (Beyotime Biotechnology, Shanghai, China), an analog of Fluo-3 AM, has been extensively utilized for measuring intracellular calcium ion concentrations in mammalian cells [32,33]. Based on these established methods, the intracellular calcium ion concentration of *C. albicans* ATCC MYA-2876 was examined. Briefly, overnight cultured fungal cells were diluted to 1 × 10^6^ cells/mL in YPD medium and 250 µg/mL Equol at 37 °C for 8 or 12 h with shaking at 200 rpm, collected by centrifugation, washed twice in sterile PBS, resuspended in 500 µL PBS, incubated with Fluo-4 AM (final concentration of 2 µM) for 40 min at 37 °C in the dark, and then washed three times with PBS. The fluorescence intensity of the Fluo-4 AM probe was measured after another 20 min of incubation at 37 °C in the absence of Fluo-4 AM. Untreated fungal cells were used as a negative control.

### 2.11. Measurement of MMP

Changes to the MMP of *C. albicans* cells after Equol treatment were assessed using a JC-1 MMP assay kit (Beyotime Biotechnology) in accordance with the manufacturer’s protocol [34,35]. The depolarization of the MMP after Equol treatment was assessed using the molecular probe JC-1. The treated fungal cells were washed with PBS and then incubated with the JC-1 probe for 20 min at 37 °C. After incubation, the cells were washed twice with JC-1 buffer, and the green fluorescence intensity of the JC-1 probe was measured at excitation and emission wavelengths of 490 and 530 nm, while red fluorescence was measured at excitation and emission wavelengths of 525 and 590 nm using a multifunctional microplate reader and fluorescence microscopy, respectively. Untreated fungal cells were used as a negative control and carbonyl cyanide m-chlorophenyl hydrazone (CCCP) was used to decrease the MMP as a positive control. Red fluorescence (JC-1 aggregates) indicates healthy mitochondria with a high MMP and green fluorescence (JC-1 monomer) indicates mitochondria with a low MMP.

### 2.12. Transmission Electron Microscopy (TEM)

Morphological changes to Equol-treated cells were observed by TEM [36]. Briefly, fungal cell suspensions (1 × 10^6^ cells/mL) were exposed to 250 μg/mL Equol and incubated at 37 °C and 200 rpm for 8 or 12 h. Untreated fungal cells were used as a negative control. The cells were collected by centrifugation at 12,000× *g* for 10 min, prefixed with 3% glutaraldehyde, postfixed with 1% osmium tetroxide, dehydrated, embedded in epoxy resin, and cut into semi-thin sections, which were stained with methylene blue, or ultrathin sections with a diamond knife and stained with uranyl acetate and lead citrate, and examined by TEM (JEM-1400-FLASH; JEOL, Ltd., Tokyo, Japan).

### 2.13. Statistical Analysis

All statistical analyses were performed using Prism 8 software (GraphPad Software, San Diego, CA, USA). A two-sided Student’s *t*-test and a one-way analysis of variance (ANOVA) were used to compare differences between and among groups, respectively. The data are presented as the mean ± standard error. A probability (*p*) value ≤ 0.05 was considered statistically significant. All experiments were repeated in triplicate.

## 3. Results

### 3.1. Equol Inhibited Growth of Fungal Cells

**Figure 1:**
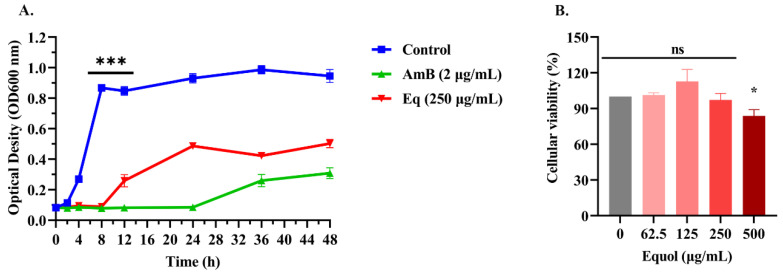
Growth curves of *C. albicans* ATCC MYA-2876 and Macrophage toxicity. (**A**) *C. albicans* ATCC MYA-2876 (5 × 10^5^ CFU/mL) treated with Equol or amphotericin B. AmB, amphotericin B; Eq, Equol. (**B**) The effect of various concentrations of Equol on the 12 h survival rate of Ana-1 macrophages was detected by using the Cell Counting Kit-8. Data were analyzed by one-way ANOVA (ns: *p* > 0.05, * *p* < 0.05, *** *p* < 0.001).

As shown in Table 1, Equol showed antifungal activity against *C. parapsilosis*, *C. neoformans*, *C. auris*, and *C. duobushaemulonii* with MIC values ranging from 125 to 500 µg/mL. A growth curve analysis showed that untreated *C. albicans* entered the logarithmic phase within 4 h of incubation and reached the plateau phase at 8 h, while *C. albicans* treated with Equol (250 μg/mL) entered the logarithmic phase only at 12 h after incubation and reached the plateau phase at 24 h (Figure 1A). Moreover, the results indicated that less than 250 μg/mL of Equol was non-cytotoxic (Figure 1B).

### 3.2. Equol Caused Ultrastructural Changes to Fungal Cells

**Figure 2:**
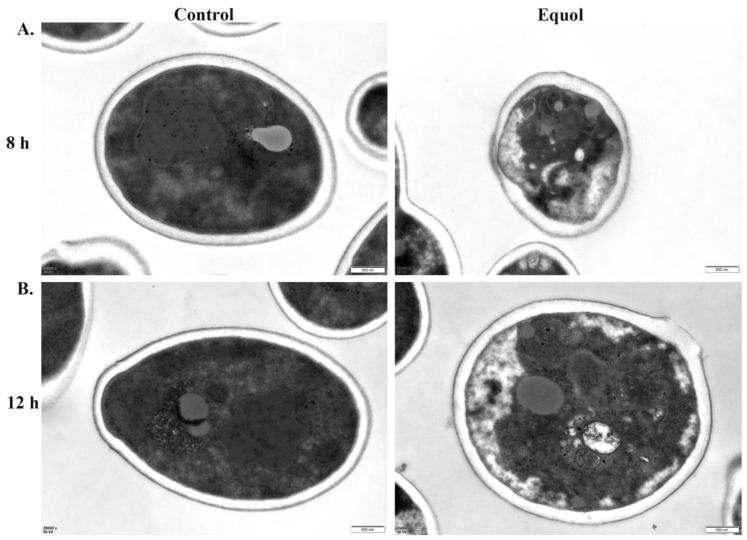
TEM results of *C. albicans* ATCC MYA-2876. *C. albicans* ATCC MYA-2876 treated with Equol (250 μg/mL) for 8 h (**A**) and 12 h (**B**). Scale bar: 500 nm.

The TEM analysis showed that the untreated cell wall, cell membrane, and cytoplasm were dense with complete structures and boundaries of the organelles. In addition, the fungal cells were round or oval with a uniform size. However, after exposure to Equol at 250 μg/mL for 8 and 12 h, the fungal cytoplasm was loose, the organelle structures were disorganized, the number of vacuoles was increased, the cell membrane was zigzagged and uneven, and the cell wall was destroyed, allowing for leakage of intracellular substances. These findings suggest that Equol caused morphological changes to the fungal cells and destroyed the surface structure (Figure 2), resulting in intracellular imbalance and inhibition fungal cell growth.

### 3.3. Equol Damaged the Fungal Cell Wall and Membrane

**Figure 3:**
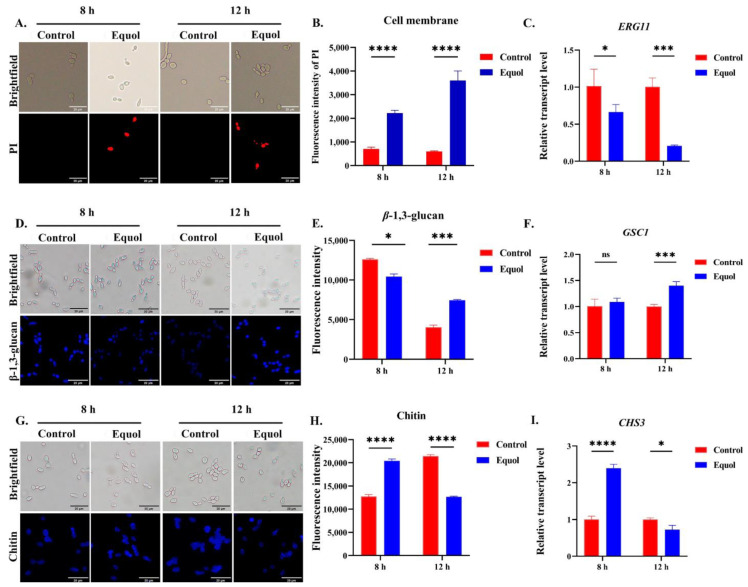
Effect of Equol on the cell wall and membrane. (**A**) *C. albicans* ATCC MYA-2876 was stained with PI to determine the extent of cell membrane damage. Observe the extent of cell membrane damage after treatment with Equol using an inverted fluorescence microscope. Bar: 20 μm. (**B**) The degree of membrane damage was measured by zymography. (**C**) Expression of the cell membrane synthesis-related gene *ERG11*. (**D**) Determination of β-1,3-glucan content using the aniline blue fluorescence staining method and photographic observation under a fluorescence microscope. Bar: 20 μm. (**E**) The content of β-1,3-gluacn was determined by zymography. (**F**) Quantitative expression of β-1,3 glucan synthase (*GSC1*). (**G**) Chitin (stained with CFW) of *C. albicans* ATCC MYA-2876 after Equol treatment for 8 and 12 h was detected with the use of fluorescence microscopy (bar: 20 μm) and by zymography (**H**). (**I**) Quantitative expression of chitin synthase (*CHS3*). Data were analyzed by one-way ANOVA and the *t*-test (ns, *p* > 0.05; * *p* < 0.05; *** *p* < 0.001; **** *p* < 0.0001).

PI enters the cell and binds to DNA with red fluorescence after damage to membrane integrity [28]. The PI staining results showed that the fluorescence intensity was stronger after Equol treatment as compared to the control group (Figure 3A,B). Further RT-qPCR analysis of *ERG11*, a key synthesis gene for ergosterol [37], revealed that Equol decreased the expression of *ERG11* (Figure 3C).

Meanwhile, fluorescence staining of the important components of the fungal cell wall, including chitin and β-1,3-glucan [29], confirmed that the cell wall components had undergone changes over different time periods after Equol treatment. Specifically, the content of the content of β-1,3-glucan had decreased (Figure 3D,E), while the chitin of the cell wall had increased (Figure 3G,H) after 8 h of Equol treatment. However, opposite results were observed after 12 h of Equol treatment. Further RT-qPCR analysis showed that the β-glucan synthesis gene GSC1 (Figure 3F) and chitin synthesis gene CHS3 (Figure 3I) were consistent with these changes. Specifically, GSC1 expression was upregulated after drug treatment for 12 h, while CHS3 expression was upregulated after drug treatment for 8 h. These findings demonstrate that Equol remodeled and disrupted the cell wall and impaired cell membrane integrity.

### 3.4. Equol Caused Intracellular ROS and Ca^2+^ Overload

**Figure 4:**
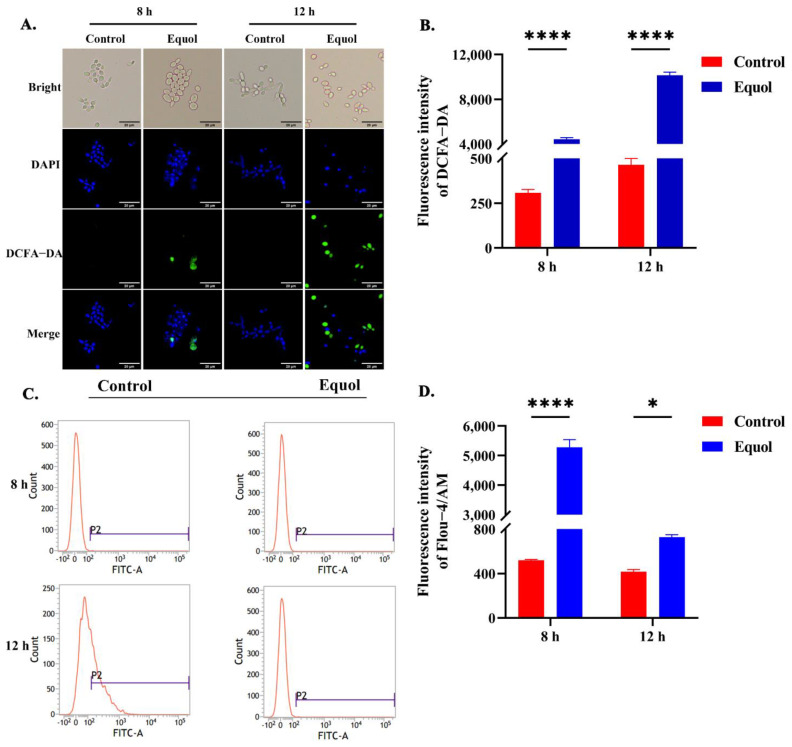
Effect of Equol on intracellular ROS and Ca^2+^ levels. Cells were incubated in the presence or absence of Equol for 8 and 12 h and then incubated with DCFH-DA (40 μmol/L). Intracellular ROS levels were assessed by (**A**) fluorescence microscopy (bar: 20 μm) and (**B**) zymography. The Fluo−4 AM probe was used to detect changes to intracellular Ca^2+^; the levels were assessed by (**C**) flow cytometry and (**D**) zymography. Data were analyzed by one-way ANOVA and the *t*-test (* *p* < 0.05; **** *p* < 0.0001).

An imbalance in intracellular ROS leads to apoptosis [38]. Therefore, alterations to intracellular ROS contents were detected. As expected, the intracellular ROS content of fungal cells had progressively increased after Equol treatment, as demonstrated by enhanced green fluorescence (Figure 4A,B). Moreover, ROS and Ca^2+^ complement each other and are considered to be crucial mediators of apoptosis [39,40]. In this study, the accumulation of intracytoplasmic Ca^2+^ was significantly increased after 8 h of Equol treatment as compared to the control group. In addition, as compared to the control group, Ca^2+^ after 12 h of Equol treatment was increased and significantly increased after 8 h (Figure 4C,D). These data indicate that the culture of *C. albicans* with Equol leads to the generation of ROS and the accumulation of Ca^2+^, suggesting that Equol causes oxidative damage to fungal cells.

### 3.5. Equol Induced Mitochondrial Dysfunction

**Figure 5:**
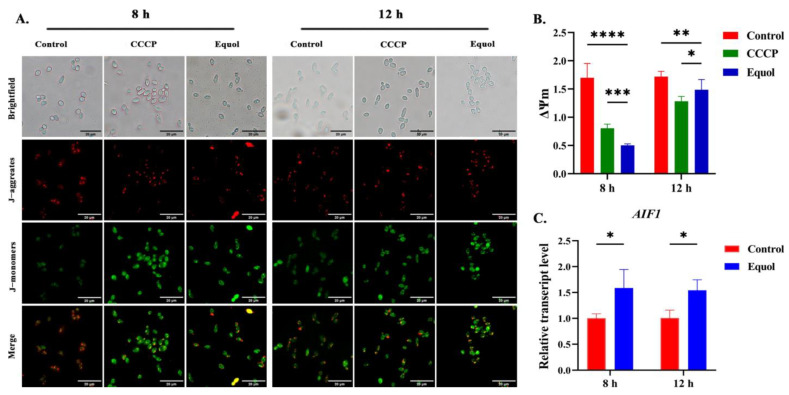
Effect of Equol on fungal MMP. (**A**) Determination of intracellular mitochondrial membrane potential levels in Candida albicans using the JC-1 probe. JC-1 aggregates emitted red fluorescence, while JC-1 monomers emitted green fluorescence. Merged images showing the extent of the loss of MMP. Scale bar: 20 µm. Carbonyl cyanide m-chlorophenyl hydrazine (CCCP) is a mitochondrial uncoupling agent as a positive control. (**B**) The bar chart shows that intracellular MMP levels were assessed by zymography. (**C**) Expression changes of the mitochondrial-related apoptosis-inducing factor gene *AIF1*. Data were analyzed by one-way ANOVA and the *t*-test (* *p* < 0.05; ** *p* < 0.01; *** *p* < 0.001; **** *p* < 0.0001).

The ROS overproduction and disruption of Ca^2+^ homeostasis induce mitochondrial damage [15]. In order to evaluate the effects of Equol on mitochondrial function of *C. albicans*, changes in MMP and the expression of related apoptosis genes were detected. The detection of MMP by a JC-1 fluorescence probe showed that compared with the control group, both the CCCP (a mitochondrial uncoupling agent)- and Equol-treated groups showed enhanced green fluorescence and weakened red fluorescence at 8 and 12 h, and the red/green fluorescence ratio of JC-1 was decreased, indicating mitochondrial membrane potential depolarization (Figure 5A,B). These results indicated that Equol damaged mitochondrial membrane potential and mitochondrial homeostasis was unbalanced. The expression level of gene-encoding mitochondrial-related apoptosis factor *AIF1* showed that the transcription level of *AIF1* was significantly upregulated after 12 h of Equol treatment, suggesting that Equol may induce apoptosis of *C. albicans* cells by activating the mitochondrial pathway (Figure 5C).

## 4. Discussion

The incidence of invasive *Candida* infections continues to increase yearly due to the rise in immunocompromised populations, as well as the misuse of antibiotics and antifungal medications, and the impact of the coronavirus disease pandemic in 2019, which has generated a large number of susceptible populations [41,42,43]. However, inappropriate antifungal drugs and the emergence of multidrug-resistant strains have led to a decline in the effectiveness of existing antifungal treatments [44]. Therefore, novel targeted antifungal drugs are urgently needed. Previous investigations by our group confirmed that Equol exerts antifungal effects against *C. albicans* by inhibiting mycelial transformation and biofilm formation, as well as destroying formed biofilms. However, the intracellular antifungal mechanism of Equol has not been thoroughly investigated [27]. The results of the present study demonstrated that Equol also has antifungal activity against non-*C. albicans* species. Further investigations on the intracellular antifungal molecular mechanism revealed that Equol can effectively destroy the fungal cell wall and membrane integrity and cause the depolarization of MMP, which collectively ultimately induce apoptosis.

The cell wall not only plays an important role in the maintenance of cell morphology and polarized growth, but also in adhesion with host cells and phagocytosis [45,46]. Previous investigations have demonstrated that antifungal drugs, such as echinocandins and β-glucan derivatives, target with the cell wall and enhance intracellular ROS levels, thereby achieving an inhibitory effect against fungal cells [47,48]. In this study, observations by TEM showed that treatment with Equol severely damaged the integrity of the fungal cell wall (Figure 2 and Figure 3). In addition to the cell wall, the cell membrane also plays an important role in maintaining and regulating the cellular structure and function [49,50,51]. The fluorescence staining results indicated that Equol also disrupted the integrity of the cell membrane (Figure 3). Therefore, the disruption of the cell wall and membrane by Equol would cause changes to fungal cell permeability and the intracellular environment. However, changes to the intracellular environment must be further confirmed.

ROS play an important role in biological processes, such as proliferation and the regulation of the cell cycle. An imbalance in intracellular ROS has been shown to lead to apoptosis in fungal cells [38]. In this study, intracellular ROS were significantly increased after Equol treatment (Figure 4). The accumulation of ROS damages cells and mediates mitochondrial dysfunction. Moreover, oxidative stress as well as mitochondrial dysfunction are important molecular targets for the action of antifungal agents. For example, the antifungal activity of kalopanaxsaponin A is due to increased intracellular ROS production [52,53]. Moreover, the antifungal effects of allotoxin A1 are due to increased intracellular ROS accumulation and mitochondrial dysfunction [54]. In addition, the antifungal effects of the peptide LL-37 have been linked to the dysregulation of Ca^2+^ homeostasis and increased ROS production in mitochondria [55]. Therefore, agents that target the fungal mitochondria provide a promising antifungal strategy.

As an important second messenger in the developmental and stress signaling pathways of fungal cells, Ca^2+^ plays an important role in cation homeostasis, morphogenesis, virulence traits, and antifungal resistance [56,57,58]. Elevated cytoplasmic Ca^2+^ content has been shown to be the main cause of pheromone-induced apoptosis in *Saccharomyces cerevisiae* [39,40,53]. Meanwhile, the Ca^2+^ and ROS signaling pathways overlap and influence each other, as both are involved in cellular stress, injury, and even apoptosis in fungal cells [59]. Therefore, both pathways are considered key mediators of apoptosis [38,60,61]. In this study, Equol treatment increased intracellular concentration of both ROS and Ca^2+^ (Figure 4). These complementary effects were mainly manifested by the accumulation of ROS, which leads to the release of Ca^2+^ from the endoplasmic reticulum or other organelles, and Ca^2+^ overload, which disrupts Ca^2+^ homeostasis in the mitochondria and increases the production of ROS [38,60,61].

Oxidative stress and Ca^2+^ overload also increase the permeability of the mitochondrial membrane and subsequent depolarization [39]. Increased permeability of the inner mitochondrial membrane also causes the release of protons, resulting in the depolarization of the MMP, which is one of the earliest stages of apoptosis [62,63]. As potential targets for the development of antifungal agents, the mitochondria are responsible for cellular adaptation to oxidative stress and Ca^2+^ overload and play an important role in the production of adenosine triphosphate, the control of lipid homeostasis, and the regulation of programmed fungal cell death [18,64]. Under normal conditions, the intracellular MMP is in a steady state, as determined by the red fluorescence of the JC-1 probe. Conversely, in a state of apoptosis, the MMP is depolarized, and the JC-1 probe emits green fluorescence. In the present study, the intracellular monomeric form of JC-1 (green fluorescence) had increased after Equol treatment (Figure 5). *AIF1* codes for a conserved flavoprotein located in mitochondria that acts as an apoptosis-inducing factor that leads to nuclear cohesion and DNA disassembly after translocation to the nucleus, ultimately inducing apoptosis [65,66]. *AIF1* expression was significantly increased after Equol treatment (Figure 5), providing favorable evidence for mitochondrial membrane depolarization and subsequent apoptosis. Moreover, it had confirmed that even within the same class of drugs, the drug sensitivity and response varied among various strains [67]. Therefore, the effect of Equol on other clinically relevant strains, such as *C. auris* or *C. parapsilosis*, still needs to be further validated.

## 5. Conclusions

**Figure 6:**
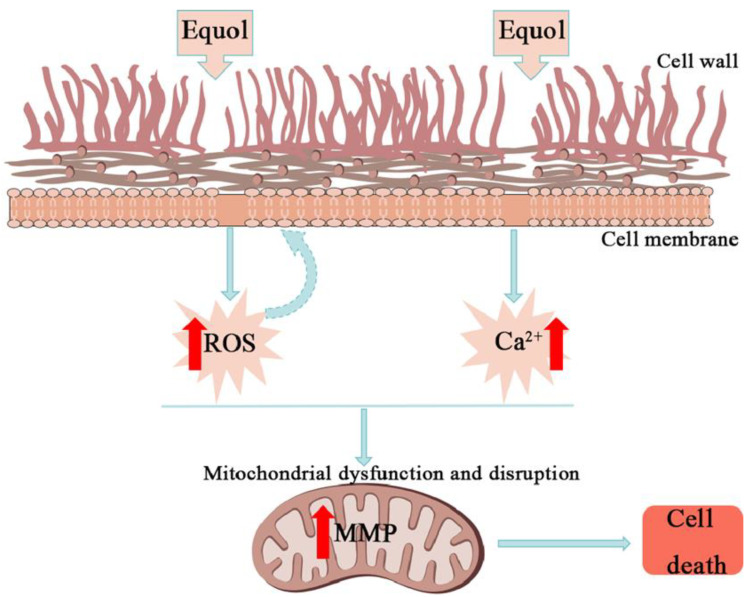
Model of the anti-*C. albicans* mechanism. Disruption of the fungal cell wall and membrane integrity by Equol caused an increase in intracellular ROS and Ca^2+^, leading to mitochondrial dysfunction. MMP: mitochondrial membrane potential; ROS: reactive oxygen species.

The results of this study confirmed the antifungal effect of Equol against *Candida* species. The mechanism underlying the antifungal effect of Equol against *C. albicans* involves significant damage to the cell membrane, cell wall, and mitochondrial membrane, along with changes to the intracellular environment, resulting in the apoptosis of *C. albicans* (Figure 6). Collectively, these findings highlight the potential clinical use of Equol as an antifungal agent.

## Figures and Tables

**Table 1:** Drug susceptibility of different fungal species.

Strains	MIC_90_ (µg/mL)	
	Equol	AmB
*C. albicans* ATCC MYA-2876	250	1
*C. parapsilosis* ATCC 22019	250	0.5
*C. neoformans* ATCC 208821	250	0.5
*C. auris* swcau	125	0.5
*C. auris* swcau1	125	0.5
*C. duobushaemulonii* swcd	500	0.5

Note: MIC_90_, minimal drug concentration that inhibits the growth by 90%; AmB, amphotericin B.

## Data Availability

All data generated or analyzed during this study are included in the article materials, which are available from the corresponding author upon reasonable request.
